# What Black Mothers with Preterm Infants Want for Their Mental Health Care: A Qualitative Study

**DOI:** 10.1089/whr.2022.0088

**Published:** 2023-02-06

**Authors:** Kobi V. Ajayi, Whitney R. Garney

**Affiliations:** ^1^ Department of Health Behavior, School of Public Health, Texas A&M University, College Station, Texas, USA.; ^2^ Laboratory for Community Health Evaluation and Systems Science, Department of Health Behavior, School of Public Health, Texas A&M University, College Station, Texas, USA.

**Keywords:** premature birth, perinatal mental health, neonatal intensive care unit, Black mothers, culturally competent care

“I think that some culturally sensitive mental health information could have been provided”: What Black mothers with preterm infants want for their mental health care: A qualitative study

## Background:

In the United States, preterm birth (PTB) rates in Black women are 50% higher than in non-Hispanic White and Hispanic mothers. Existing discriminatory sociohistorical and contemporary health care practices have been linked to the alarmingly higher rates of PTB among Black families. While it is well-known that PTB is associated with increased mental health (MH) problems, Black women experience elevated MH burdens due to inequities along the care continuum in the neonatal intensive care unit (NICU). Consequently, culturally responsive MH care holds promises to achieve maternal MH equity. This study aimed to explore the available MH services and resources in the NICU for Black mothers with preterm infants. We also sought to discover potential recommendations and strategies for MH programs through a cultural lens.

## Materials and Methods:

Semistructured interviews were conducted with Black mothers with preterm infants using a Grounded Theory approach embedded in the Black feminist theory.

## Results:

Eleven mothers who gave birth to a preterm infant between 2008 and 2021 participated in this study. Eight women reported not receiving MH services or resources in the NICU. Interestingly, of the three mothers who received MH referrals/services, two did so one-year postbirth and did not utilize the services. Three main themes emerged: stress and the NICU experience, coping mechanisms, and culturally appropriate MH care with diverse providers are needed. Overall, our finds suggest that MH care is not prioritized in the NICU.

## Conclusion:

Black mothers with preterm infants encounter numerous negative and stressful experiences that exacerbate their MH during and beyond the NICU. However, MH services in the NICU and follow-up services are scarce. Mothers in this study endorsed creating culturally appropriate MH programs that addresses their unique intersections.

## Introduction

It is a well-known phenomenon that having a preterm birth (PTB)—a baby born before 37 weeks of complete pregnancy, is associated with increased mental health (MH) problems, including posttraumatic stress, depressive symptoms, anxiety, and worry symptoms in mothers.^1–4^ MH problems arise from numerous factors within and beyond the neonatal intensive care unit (NICU) hospitalization and are experienced by most mothers regardless of race and ethnicity.^2,3^ However, these experiences are magnified for Black mothers due to discriminatory health care practices, excess psychosocial stress burden, and structural disadvantages, mostly rooted in racism, leading to greater MH burdens.^5–9^ Despite their vulnerability, there are racial inequities in MH care and treatment access.

For example, Black women are more likely to report receiving hospital-based postpartum depression care instead of referral to psychiatrists and stigma regarding their care than white women.^10,11^ As a result, there is no shortage of call for MH programs and policies that are culturally appropriate and considers the intersections of Black pregnant and postpartum mothers, thereby achieving maternal health equity.^12,13^

In the United States, although the rates of PTB among Black women are 50% higher than in White and Hispanic mothers, research suggests significant health disparities related to the process, structure, and outcome of NICU hospitalization, which disproportionately affects Black mothers and infants.^14–16^ For example, Black families are more likely to be segregated to lower-quality NICU hospitals and not receive family centered-care in the NICU.^17,18^ This ostensibly contributes to the increased Black maternal and infant morbidity and mortality in the United States.^19,20^ Indeed, disproportionate NICU care is undeniably a catalyst for poor MH in Black mothers and at the core of these disparities is a lack of culturally competent health care practices that center on voices of Black mothers in their care.^13^

Consequently, there is an urgent need to ground the MH care of Black mothers with PTB through cultural and intersectional lenses. According to the national standards for culturally and linguistically appropriate services in health and health care developed by the Office of Minority Health of the U.S. Department of Health and Human Services, culturally “responsive,” “targeted,” or “tailored” care are services that are respectful of cultural beliefs, practices, and needs of diverse and minority populations.^21^ Research has shown that culturally responsive care effectively bridges racial/ethnic disparities among ethnic minority people.^22–25^ Because culture is a critical determinant of health, incorporating aspects of one's culture in their care can improve patient-centered outcomes and is more effective than a one-size-fits-all approach.^25^ Cultural adaptations can be done through several strategies, including peripheral, evidential, linguistic, constituent-involved, and sociocultural, and are effective if used independently or combined.^25^

However, despite the utility of culturally responsive interventions and the increased MH burden among Black mothers with PTB, little research has sought to understand their MH needs.^26^ Previous NICU-based psychological interventions primarily use a one-size-fits-all approach that undermines the intersections and sociocultural idiosyncrasies that affect Black mothers.^27,28^ Other studies have focused on other health care needs of pregnant and postpartum women of color.^13,29–31^ As a result, investigating the MH needs of Black mothers with PTB is critical to achieving equitable maternal and infant health outcomes. Therefore, this study aims to explore available MH services and resources in the NICU for Black mothers with PTB. Secondarily, this study sought to discover potential recommendations and strategies for MH programs through a cultural lens.

## Research Questions

This study answers the following research questions:

1. What are the available MH resources and services in the NICU?
2. How do Black mothers with PTB in the United States describe their MH care experience in the NICU?
3. What strategies do Black mothers with PTB in the United States recommend in developing a culturally tailored perinatal depression prevention program for Black mothers with PTB?

## Materials and Methods

### Study design

This study is a qualitative exploratory inquiry based on grounded theory (GT). GT systematically and synergistically collects data to generate new theories “grounded” in the data and when there is limited knowledge about the phenomenon.^32^ Due to gaps in the literature about the MH programs in the NICU, GT is more appropriate because it aids researchers in producing new theories to design culturally relevant MH programs for Black mothers with PTB. In addition to GT, this study is embedded in the theoretical ideals of Black feminist theory. The Black feminist theory recognizes the historical and contemporary racism experienced by Black women and works to reclaim their power by centering their voices and experiences.^33^ Together, these two theories underscore the need to advance maternal health equity in the United States by including Black mothers in the research process to generate new approaches to health interventions.

A semistructured interview was used to generate data for this study, and the data were analyzed through thematic analysis and the GT framework. All Interviews were conducted virtually via Zoom.

### Recruitment and sampling

Convenience and snowball sampling were used to recruit participants. Fliers were posted on several social media platforms. Specifically, fliers were posted on a closed Facebook group: Black Ladies in Public Health (BLiPH). BLiPH is a registered nonprofit organization in the United States comprising Black/African American women working broadly in fields that promote the health of Black women and their communities. The group contains more than thirteen thousand members and includes undergraduate and graduate students and professionals working in diverse settings (https://www.facebook.com/groups/BLiPH). Posting the fliers in this group is ideal because of the group's demographic. Moreover, research indicates that involving Black mothers in the research process is critical to creating culturally responsive care.^33,34^

To be included in this study, mothers had to (1) be 18 years of age or older, (2) self-identify as Black/African American with PTB in the United States, (3) have had an infant NICU hospitalization, and (4) ability to speak English. We did not restrict our sample to a time range to allow us to capture the mothers' temporal experiences if reported. In the same vein, there were no geographic restrictions. We excluded mothers with term babies (i.e., above 37 weeks gestation) with a NICU hospitalization and those without a NICU hospitalization not directly attributed to PTB. Participation was voluntary, and written and oral informed consent was obtained from each participant.

### Data collection guide

In line with the research paradigms used in this study and culturally responsive care framework,^32–34^ mothers with PTB and professionals who provide services in various capacities from the BLiPH group were invited to provide initial recommendations for the interview guide. Soliciting the viewpoints of individuals from the BLiPH group in developing the interview guide aligns with the Black feminist theory mindset by centering the voices of Black women in developing and testing research hypothesis and solutions versus research subjects only. The recommendations received from BLiPH were triaged with the literature to develop the interview guide.^29^ The guide was then iteratively reviewed between the first author and another researcher with extensive experience in women's health research among racially and ethnically diverse populations.

A final interview guide was approved and subsequently used for this study, which contained sociodemographic questions and a battery of questions related to different domains of NICU care. All interviews were conducted between September and November 2021 by the first author via Zoom until no new information was forthcoming and data saturation was achieved. The interviews were audio recorded and transcribed verbatim using a secure and approved transcription service. In addition to the guide, field notes were written after each interview to capture salient aspects (e.g., voice, mood, and other thoughts) that may not have been captured in the interview process. The interviews lasted for ∼1 hour.

This qualitative study is part of a larger ongoing mixed-method study aimed at developing a culturally appropriate postpartum depression program for Black mothers with PTB.^26^ However, data for this study were pooled from the MH questions of the qualitative study (Supplementary Tables S1 and S2).

### Data analysis

Data were analyzed by the first author using the GT and thematic analysis framework. The codebook was developed inductively based on Decuir-Gunby et al's data-driven code generation approach,^35^ which aligns with the GT framework. At first, the interview data were read in its entirety to enable the familiarization with salient points of the data, then subsample themes were identified. Themes were then compared across subsamples before arriving at a final set of codes. In keeping with the data-driven code strategy, themes were identified by “lumping” and “splitting” text in the line, sentence, and paragraph levels to label and reduce the data based on the “level of meaning.”

To establish rigor, one researcher who was not privy to the data collection process was invited to code 5% of the interview data using the codebook. Few changes were made to the final codebook based on consensus between the two raters. Subsequently, the first author coded the remaining data based on the final codebook, allowing consistency throughout the coding process. Both raters agreed on the final themes generated.

### Ethical considerations

The study was conducted in line with the Helsinki ethical standards and approved by and in compliance with the Texas A&M University Institutional Review Board.

## Results

### Study characteristics

A total of 11 (*n* = 11) women who identified as Black individuals and had given birth to at least one preterm infant between 2008 and 2021 participated in this study. Participants ages ranged from 25 to 44; educational levels ranged from a college degree to a graduate degree and higher; income levels ranged between $35,000 and $75,000 and above. All the mothers were employed and used prenatal care during their pregnancy. See Table 1 for full participants' characteristics.

**Table 1.** Participant Characteristics

Variable	*N*	%
Age		
25–34	5	45
35–44	6	55
Marital status		
Married	9	82
Single	2	18
Annual household income		
Less than $49,999k	2	18
$50k to $74,999k	3	27
$75k and above	6	55
Educational status		
College degree	2	18
Graduate degree or more	8	73
NA^a^	1	9
Employment status		
Employed	11	100
Previous pregnancy history^b^		
No	2	18
Yes	9	82
Prenatal care use		
No		
Yes	11	100
Type of delivery		
Vaginal	2	18
Cesarean section	9	82
History of preterm birth		
No	8	73
Yes	3	27
Type of birth		
Singleton	8	73
Multiple	2	18
NA	1	9
Gestational week		
27–29 weeks	3	27
30–36 weeks	8	73
Year of birth		
2008	1	9
2010	1	9
2012	1	9
2017	1	9
2019	4	36
2020	1	9
2021	2	18

^a^ Participants did not reveal this information.

^b^ Include gestational diabetes, hypertension, and other pregnancy-related health conditions.

The themes from the interview reflected similar viewpoints regarding drivers of stress and MH problems surrounding NICU hospitalization. Although we found that mothers' MH support was overwhelmingly lacking, even though participants exhibited and struggled with poor MH, it was not particularly related to race. In contrast, it was a complete lack of MH services in the NICU. Thematic analysis revealed three dominant themes: stress and the NICU experience, coping mechanisms, and culturally appropriate MH care with diverse providers.

### Stress and the NICU experience

#### Support

Participants generally agreed that poor support from family, providers, and the NICU staff was a significant source of stress. Some mothers lived far away from their immediate family, or their families had to go to work. Others were the first in their family ever to have a preterm infant and, as a result, left to figure out how to care for their infants by themselves. Mothers also reported a lack of emotional support, lactation support, and a lack of clear and understandable information from providers made the NICU stay stressful. However, when support was offered, it was received from an unlikely source (Supplementary Table S2, Quotes 1–4).

#### Balancing life

Mothers described feeling stressed from balancing competing life demands in the NICU and after discharge. These conflicts stemmed from parenting, home management, keeping up with doctor's appointments, and work responsibilities. One mother, in particular, had to quit her job because of challenges managing her multiple roles: caring for her infant, working, and parenting her toddler while also keeping specialty appointments. Some mothers who were students and worked as full-time employees noted that it was hard to balance their roles because they were overwhelmed with thoughts of coursework and other work responsibilities. Similarly, those with toddlers noted that parenting a toddler and caring for their preterm infants was an overwhelming experience (Supplementary Table S2, Quotes 5–8).

#### Uncertainty

Uncertainty related to infant health, changes to birth plans, and the future physical health of the infant were sources of stress for mothers. A mother with a traumatic birth experience indicated that sudden changes from her birth plans as a result of having a preterm infant were stressful. Meanwhile, the uncertainties from an unexplained PTB among mothers who had seemingly smooth pregnancies without any existing health issues made the birthing experience stressful. In some cases, mothers were stressed more because of the lack of awareness about PTB before becoming mothers. They were oblivious about caring for a PTB infant or knowing what to expect about their care. One mother whose infant was sensitive to milk discussed how she was uncertain what to do because she received conflicting recommendations from providers about the best feeding options to adopt (Supplementary Table S2, Quotes 8–10).

#### Feeding challenges

Mothers reported being stressed from challenges with having an inconsistent breast milk supply. One mother had to supplement her breastmilk with baby formula because her infant was not gaining the appropriate weight, which was stressful and elicited worry. Others were worried and stressed because it was challenging to continue breastfeeding beyond the NICU (Supplementary Table S2, Quotes 11–12).

#### Triggers in the NICU environment

Mothers responded to questions about how the NICU environment affected their MH. They highlighted the sounds and lights from machines in the NICU, information overload in the NICU, or triggers from the routine that made them sad and anxious. One mother noted that malfunctioning equipment in the NICU led to “up and down moments,” which triggered emotional reactions and anxiety. However, some mothers indicated that some aspects of the NICU environment positively affected them (Supplementary Table S2, Quotes 13–14).

#### Other experiences

Beyond the stress of having a PTB, mothers with preexisting trauma from previous PTB delivery and childhood trauma, unplanned pregnancy, and the impact of the COVID-19 pandemic also compounded the stress they felt (Supplementary Table S2, Quotes 15–17).

### “I just gave that up”: Coping mechanisms

#### Calling yet unheard

**FIG. 1.**
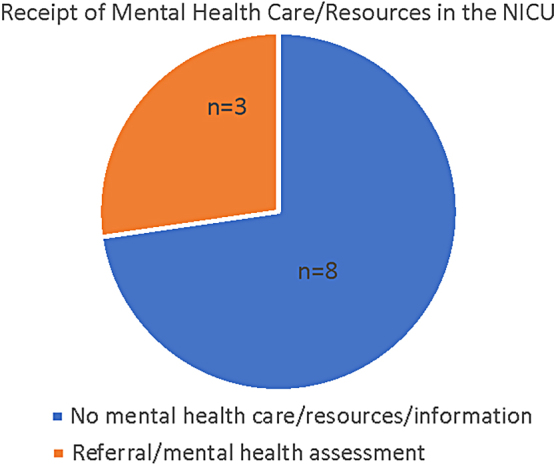
Pie chart showing the receipt of mental health care/resources in the NICU among Black mothers. NICU, neonatal intensive care unit.

Since an overwhelming number of mothers (*n* = 8, Fig. 1) did not receive any MH resources, including referrals to psychiatrists and/or chaplain services, community resources, or even discussed issues about their mental and emotional well-being, it became apparent that they needed to figure out how to manage their MH by themselves. Those who verbally requested MH care were not provided any support because the hospitals did not offer MH services for the NICU (Supplementary Table S2, Quotes 18–19).

#### Available but not accessible

Of the mothers who said they received MH resources such as filling out MH assessment questionnaires, referral to hospital-affiliated support groups, or a social worker, only one mother indicated that they engaged the social worker who talked to her about her feelings. The others could not keep up with the programs because of time constraints. Interestingly, two mothers who received MH referrals to a local support group only received the information a year after their infants were born (Supplementary Table S2, Quote 20).

#### Figuring it out

As a result of the lack of services, mothers independently sought diverse avenues to address their MH needs through prayers, informal support, being mentally prepared based on previous PTB experiences, seeking therapy, and centering themselves by reframing their mindset to focus on their family and self. In contrast, others “just gave up” on activities that did not prioritize their MH, even if it would cost them providing breastmilk to their infant (Supplementary Table S2, Quotes 21–24).

One mother who gave birth during the COVID-19 pandemic lockdown noted that her employer was very understanding and supportive by providing paid leave for people who have to care for kids during COVID-19. Regarding whether mothers believed that the NICU should offer MH care and if they would have utilized those services while in the NICU, mothers unanimously agreed that there should be an in-house therapist or staff to continually check up on mothers as they would have utilized such services were they available.

Field notes retrieved from the interview revealed that mothers thought the NICU staff only focused on the infant's health at the expense of the mothers. As a result, they felt unheard and unseen even as they struggled to care for their infants.

### Culturally relevant MH care with diverse providers is needed

Ideas shared by participants reflect what Black women, in general, want concerning their MH needs with recommendations more relatable to the NICU. Mothers endorsed a holistic MH program specific for mothers (but could include other women of color) that combine informal (e.g., other Black mothers with similar lived experiences of having preterm infants, Black doulas, family members) and formal support from diverse health care providers (e.g., Black therapist, lactation consultant). They also suggested that a support group format may be appropriate to lean into the support from others with similar experiences.

However, they opined that researchers must be cognizant of the context to know when a group or individual program would be appropriate. Participants embraced the idea of a family-centered support program that includes family members such as dads, partners, or significant others but cautioned that such programs should be designed in such a way that features mothers-only sessions, family members-only sessions, and group sessions. All the mothers stressed the need for inclusive languages that are culturally sensitive to Black mothers and address the sociohistorical aspects of their lives.

Furthermore, participants shared that such MH programs should incorporate virtual components that they can utilize at their own time and pace, considering their conflicting schedules. They also reiterated the importance of such a program being affiliated with a hospital to encourage participation. Finally, mothers approved of providing practical incentives such as transportation, internet access, childcare, meditation, yoga passes, or affirmations to promote the acceptance of MH services. Some of the perspectives shared can be seen in Supplementary Table S2, Quotes 25–28.

## Discussion

This study revealed several narratives about MH care for Black mothers with preterm infants with NICU hospitalization. First, mothers generally agreed that the NICU experience was a major determinant of stress further compounded by other external factors outside the NICU. Second, notwithstanding the temporal differences in the period of birth (i.e., 2008 to 2021), mothers revealed an overwhelming lack of MH resources or services in the NICU, even though they were visibly emotionally unstable in most cases.

The mothers reiterated that NICU staff and providers only focused on “saving” their infants without considering their physical or emotional well-being. As a result, their MH suffered greatly, which unfortunately was exacerbated by previous MH problems or life situations. Third, participants agreed that a culturally sensitive MH program (support group or curriculum-based) is highly needed to address MH issues. Importantly, they echoed that such MH programs should include diverse practitioners (e.g., nurses, therapists, and peer support) and be offered by people who look like them and share experiences of being a Black person and having a PTB.

The mothers in this study perceived their NICU experiences as largely stressful due to multifactorial events within and outside the NICU. Our findings add to a growing body of research that having a PTB induces poor MH.^26^ The participants outlined specific examples of how having low formal and informal social support, breastfeeding challenges, balancing competing life demands, concerns about the survival of their infants, and previous traumatic experiences contributed to the stressors they encountered. These divergent experiences suggest that a one-size-fits-all approach to addressing stress in the NICU may not be feasible. Instead, future research, interventions, and policies should use a collaborative process that integrates the voices of pregnant women of color, diverse providers, and the community.

Furthermore, future research should consider expanding NICU intervention research to Black birthing people instead of mothers and their partners alone to allow for more gender expansions in this area, given that MH problems are pervasive among gender and minority Black people.^36^

In addition, continuity of care, follow-up programs, safety-net community-based programs, and referral resources geared toward post-NICU hospitalization must be considered since the experiences mothers encounter transcend beyond the NICU.^37,38^ Mothers noted that negative experiences arose from feeling unheard and unable to contribute to their health care decision-making process. As such, our findings expand on the literature on the need for quality patient-provider relationships and efforts to advance family-centered NICU care that incorporates the perspectives of Black women in their care and for providers to actively listen to them.^30,39^ Moreover, considering the unique experiences of Black mothers, future research, programs, and policies should consider an intersection lens as they could help ensure that their needs are heralded, thereby improving the quality of care.^33^

Due to a lack of MH resources, mothers in this study had to seek help independently through several approaches. They mentioned using therapy, participating in religious activities through prayers, and focusing on deliberate and intentional self-care, while others did nothing. This finding comes despite expert recommendations calling for integrating MH professionals and screening apparatus in the NICU to improve psychological functioning and overall mental and emotional well-being.^40,41^ Thus, our result calls for urgent research and multilevel health system interventions to incorporate MH services and programs in the NICU. A feasible approach to doing so can be seen in the light of the recommendations provided by mothers in this study which involves the use of diverse stakeholders, including mothers, hospital administrators, medical staff, and MH specialists.^41^

Furthermore, the need to ensure a culturally competent MH workforce must be emphasized to facilitate culturally responsive care and encourage Black women to engage with these services, thereby achieving quality and equitable care.

Simultaneously, efforts to leverage nontraditional MH care (e.g., community-based organizations, faith-based organizations, peer support, or doula care) hold promise to address the MH needs of Black women and can be incorporated into the health system.^13,42,43^ Still, linking mothers with existing resources that address other socioeconomic risk factors that increase their susceptibility to poor MH should be considered.,^37^ Importantly, our study demonstrates that improving MH care in the NICU requires that systems and structures are put in place to dismantle racial inequities in access to MH programs from a holistic standpoint.

Finally, mothers in this study expressed their approval for culturally responsive MH care spearheaded by people who “look like them.” These findings support previous research outlining that women of color want and need culturally responsive and concordant care for improved health outcomes.^29,30^ Yet, our examination of the perspective of Black mothers with PTB about their recommendations on the MH care they want is novel, as these perspectives have not yet been explored. Although we did not directly measure racism or discrimination, the mother's recommendations were rooted in discriminatory health care experiences due to racial/ethnic identities.

As a result, our work sets the stage for future work to address Black maternal MH at the intersection of racial and ethnic identities/experiences and the NICU, indicating the imperative for culturally appropriate MH NICU interventions. For example, previous research has reported the effectiveness of MH interventions across different spectra (e.g., depression, anxiety, post-traumatic stress disorder) for families in the NICU.^44,45^ However, these studies are limited in the diversity of research subjects. As such, we advocate for culturally designed interventions for Black mothers given their intersecting identities, leading to excess MH burdens.

A major strength of this study is that it expands the knowledge on MH care for Black mothers with PTB to further the discussion and base future qualitative and quantitative research through strong research paradigms and frameworks. However, the findings should be interpreted in light of some limitations. First, the sample size, localization of the study to the United States, and health care context may skew the generalizability of our findings. Second, because of the study's aim, mothers from other racial/ethnic backgrounds were not included in this study. As a result, we cannot infer comparisons of the experiences and recommendations on MH care among other groups, although the findings can be useful for researchers in understanding how to develop culturally responsive MH programs for other racial/ethnic groups.

In addition, only mothers' perspectives were showcased. Ideally, balanced viewpoints that include the views of fathers, partners, and health care providers would elicit other nuanced experiences that moderate the NICU experience. Although our findings infer racially motivated care, we did not explicitly ask mothers about past or present racial discrimination, suggesting the need for further research using qualitative approaches and psychometric instruments measuring racial discrimination in the NICU setting. Finally, our sample comprised college degree earners and above that may not be necessarily socioeconomically disadvantaged. Thus, it is likely that experiences and findings would have differed were low-income mothers included in this study. Despite these limitations, our findings are a stepping stone for future research.

## Conclusions

Using the GT framework and a Black feminist mindset, we found an overwhelming lack of MH services in the NICU. Black mothers endorsed MH programs in the NICU that are culturally appropriate and incorporate diverse health care providers and other mothers who have had a previous PTB. Mothers also reported diverse stressors within and outside the NICU that negatively affects their MH. The narratives from this study call for structural- and individual-level interventions that address multifaceted aspects of the NICU experience. Importantly, this study has implications for culturally competent patient-centered care to improve the MH of Black pregnant women and birthing people.

## Supplementary Material

Supplemental data

Supplemental data

## Abbreviations Used

BLiPH: Black Ladies in Public Health
GT: grounded theory
MH: mental health
NICU: neonatal intensive care unit
PTB: preterm birth rates
