# Supplementary material for: What Black Mothers with Preterm Infants Want for Their Mental Health Care: A Qualitative Study
Source: Womens Health Rep (New Rochelle). 2023 Feb 6;4(1):39–47. doi: 10.1089/whr.2022.0088 (PMC9986014; doi:10.1089/whr.2022.0088)
Supplement: Supplemental data [file Suppl_TableS1.docx]

Supplementary Table 1: Interview Guide and Sample Questions

| **1. Stress/Mental Health Support in the NICU** | | |
| --- | --- | --- |
| 1.0 | Thinking back since your baby was born, were there times when you felt like you were completely overwhelmed? | Probe: Can you give us some examples?  Probe: What did you do when you felt overwhelmed? |
| 1.1 | Do you think you felt overwhelmed as a result of having a preterm baby? | Probe: Tell us how so? |
| 1.2 | In what ways did the NICU environment influence your mental health? | Probe: Do you think you felt more sad or anxious when you were in the NICU? |
| 1.3 | Did your NICU team help you with resources for your mental health? | Probe: What did they tell you?  Probe: Did you understand the information? |
| 1.4 | Was there anything else in your life besides having a baby in the NICU that was stressful for you? |  |
| 1.5 | Do you have any comments or thoughts about mental health resources that might have helped you as a Black woman? |  |
| **2. Logistics of a mental health intervention program** | | |
| 2.0 | If we were to design a mental health program that effectively helps Black mothers with preterm infants, describe what it might look like. |  |
| 2.1 | How would we go about it? | Probe: What type of program do you think would work: group, individual counseling, telephone, internet, blogs, etc. |
| 2.2 | When would it be best to offer this to mothers? | Probe: What are examples of the best times for this program: evenings, daytime, before the baby is born, or after birth? |
| 2.3 | Who might attend? | Probe: Examples of who should be invited to attend: other non-Black preterm mothers, dads, partners, children, immediate family members, etc. |
| 2.4 | Where is the best location? | Probe: Examples of where they would prefer to have the intervention, face-to-face at the hospital, face-to-face at home, virtual, etc. |
| 2.5 | Who should deliver this intervention? | Probe: Examples of experts that can lead the intervention, Black mothers, other mothers with preterm infants, nurses, lactation support, peer support, counselors, etc. |
| 2.6 | What are other practical cultural concerns? | Probe: Examples of things that program participants may need: childcare, incentives, transportation, electronic reminders, internet access, etc. |
