# Supplementary material for: What Black Mothers with Preterm Infants Want for Their Mental Health Care: A Qualitative Study
Source: Womens Health Rep (New Rochelle). 2023 Feb 6;4(1):39–47. doi: 10.1089/whr.2022.0088 (PMC9986014; doi:10.1089/whr.2022.0088)
Supplement: Supplemental data [file Suppl_TableS2.docx]

Supplementary Table 2: Themes, sub-themes, and exemplar quote

| Themes | Sub-themes | Exemplar Quote |
| --- | --- | --- |
| Stress and the NICU experience | Formal and informal support | *“a lot of times, we are away from family or family’s still working. My mom worked full time and lived a couple of hours away. So, I didn't have any other women per se, and my friends worked, my neighbors, and pretty much everyone I know has a full-time job...I sit here all day by myself, and if I have a question, I'm on Google because I don’t know what to do with the baby." (Quote 1).*  *“...this was the first premature baby in our family. So, like we don't know, no one knew about NICU at all...the whole experience was very isolating and lonesome, as a woman, like my mom, couldn’t help with that." (Quote 2).*  *"I just felt like I had more questions than answers. I didn’t know what to do, didn’t know who to contact; I didn’t know much of anything." (Quote 3).*  *"It was a nurse intern, a student nurse intern, that provided me some comfort and support throughout that time." (Quote 4).* |
|  | Balancing Life | *"I will say, when I returned back to work, I was overwhelmed. Because I just couldn't figure out how to balance everything. You know, I thought I was doing good, but I wasn’t." (Quote 5).*  *"After coming home, they [twin preterm infant], you know, they were on their schedules, and it was two of them, two different schedules, sometimes two different sleeping schedules. So trying to figure out when I actually needed to sleep and be productive during the day was very difficult. So that was very overwhelming for me." (Quote 6).*  *" I mean, feel overwhelmed. I had a toddler at home, trying to go back and forth to the hospital. I couldn’t drive myself. " (Quote 7).* |
|  | Uncertainty | *" It was upsetting at first. I think I was probably a little -- um, not depressed, but like baby blues for probably a month or so afterward because it was traumatic...I don’t think it was so much about him being early, but it was that I had a C-section that I wasn’t expecting. And so, after me being in labor for so long and thinking that I was going to have a regular birth this time, it was upsetting." (Quote 8).*  *"I mean like everything was done and nothing, like none of my doctor's visits, were cause for alarm, I didn't even have nausea or vomiting during my pregnancy at all...Like everything just kind of changed in a couple of hours, and we're just standing there like, “What just happened?” So that was the most stressful time, probably, in my life." (Quote 9).*  *" I didn’t know anything of it [PTB] at all until I was there, and I thought that was pretty disturbing, that how do we not talk about this. You talk about birth and pregnancy. You talk about everything as if everything goes normal all the time. We don’t talk about the complications or the possibility of changes. There are endless possibilities, and I’m the type of person who needs to know. I’d rather be prepared than unprepared. So, it was like, why don’t we talk about the things? Everything is not sunshine and rainbows all the time. Sometimes there are cloudy skies; sometimes, there’s a thunderstorm. We got to talk about those things so we can get through those things." (Quote 10).* |
|  | Feeding Challenges | *"In the hospital, he’s asleep all the time. But then you get home, and it’s like, he’s not asleep, he’s up, he’s crying. He is -- like I said, breastfeeding was hard. That was overwhelming, especially when they tell you -- he had acid reflux, and you wanted me to feed him every two hours, change him, sit him up for 10 minutes after he finished eating so he could get it and lay straight down, lay him down. You wanted me to pump for 20 minutes, and then I have to clean the pump and store the milk, and then I get to lay down. By then, it’s like, I get to lay down for maybe an hour before he’s back up and then everything. So, that’s overwhelming. I think putting that expectation on new mothers is unfair." (Quote 11).*  *"The stress of not being able [to breastfeed]-- I had difficulty breastfeeding, so that was another stressor because I couldn’t get my milk to produce. I don’t know if it was the medications or not having him at home." (Quote 12).* |
|  | Triggers in the NICU | *"I remember moments, if any time if I had to go up there, we don’t have to go hardly ever now, thank the Lord, but when we did, even the smell of the soap used to trigger me. Because I used to have to wash my hands all the time, and it would trigger my thoughts about the hospital every time I had to use it. " (Quote 13).*  *"I think being able to go and see him helped my mental health because you don’t know what's happening when you're not there, then you just able to put eyes on him and be there felt calmer." (Quote 14).* |
|  | Other experiences | *“...in many ways, I [she] was still mourning my previous birth because it happened, everything was very parallel like their due dates...I was still really grieving and mourning and just being traumatized from that. And so, I think dealing with the new baby, I’m so happy and excited, but also dealing with either grief or guilt or whatever, that loss will play the part, and I think whatever I was experiencing during that time." (Quote 15).*  *"And when that lady came and told me, you’re pregnant; I just broke down crying like, what? I’m not supposed to be pregnant. I already have a baby, and he was a preterm baby, and you telling me I’m pregnant again?" (Quote 16).*  *"[it] was stressful trying to make sure that we are on top of our health [becuase of COVID-19] so that we can maintain going to the doctor and going to the NICU every day because we didn't want to miss any time seeing him." (Quote 17).* |
| “I just gave that up.” Coping Mechanisms | Calling yet unheard | *"...I had asked the doctors about that [mental health resources]...but I asked ---- do you all know of any resources or like support groups that I can reach out to to get support for people who have had preterm babies or been through an experience like I have? Or does the hospital offer anything for parents like me? And they said the hospital doesn’t offer anything, but there is -- It was like, there is such and such organization that you can look up and go find out. " (Quote 18).*  *"That’s the biggest problem. There were none [mental health resources]. When I say none, none... And I knew there were times that I was visibly emotional. But no one asked anything. No physician asked anything." (Quote 19).* |
|  | Available but not accessible | *"They did not do it until a year later. I know that sounds crazy, but they did not give any reason. They gave me information about a local group that I couldn’t even go to anyway because I’m an hour and 30 minutes away, but it was a group of ladies that really could just help with a lot. They do self-care and have somebody to talk to for all the things I was going through with my baby." (Quote 20).* |
|  | Figuring it out | *"I figured out that I have to reframe everything, re -- or reprioritize, like, and family is first. My child who cannot care for themselves will be first. So, if it’s work or if it’s school, I’m gonna have to put that to the side and figure that out later. " (Quote 21).*  *"I stopped pumping in the middle of the night. I just gave that up. my birth companion, she wasn’t happy, she’s like, “You need to pump.” And I was like, “I can’t.” I said, “For the sake of my sanity, I cannot wake him up every two hours to feed him, change him, set him up for 10 minutes, lay him down, pump for 20 minutes, store milk, clean bottles, and then try to lay down myself.” I said, “It’s just not going to happen.” So, I made that decision just to stop pumping in the middle of the night." (Quote 22).*  *" I just tried to remind myself that I’ve had this experience before with my first child within the NICU. So, I knew that he would come home eventually, and it wouldn’t always be like this." (Quote 23).*  *"So it was overwhelming, but what helped me was prayer. And I’m not just saying, it really did, prayer and just people praying for me because there were times I couldn’t pray for myself, so just the people and my parents were there every step of the way. " (Quote 24).* |
| Culturally relevant mental health care with diverse providers is needed |  | *“I think it should be ran by mental health [providers] since that’s the focus of addressing the mental health, but I think members of the team should cover a wide range of the services offered or care that you might come into. So, like an OB-GYN, a maternal-fetal medicine doctor, a midwife, and a peer support. I think that would because it helps eliminate silos.” (Quote 25).*  *“I think having virtual makes so much sense for the rest of time… because if we continue to not have virtual options, there are so many people who just can’t make things.” (Quote 26).*  *“if it’s just for Black or African-American women, I think it will mainly just be understanding their barriers, their challenges of having a preterm birth, and how resources could behelp assist them during their journey of helping their child and making sure that their child is healthy.” (Quote 27).*  *“And I think in terms of make-up of, I think a group setting with moms of all races may be helpful, but at the same time, I think because you will have the cultural, whereas some people may not feel comfortable talking about some of the cultural things in front of people from other cultures, they may feel like, “Well, they're not going to understand what I'm saying. I feel more comfortable saying this to somebody who's going to get my story about what my aunt said.” Some of the things that happen in our families and families of color are completely different. (Quote 28).* |
